# The Parkinson’s disease waiting room of the future: measurements, not magazines

**DOI:** 10.3389/fneur.2023.1212113

**Published:** 2023-08-21

**Authors:** Jay L. Alberts, Umar Shuaib, Hubert Fernandez, Benjamin L. Walter, David Schindler, Mandy Miller Koop, Anson B. Rosenfeldt

**Affiliations:** ^1^Department of Biomedical Engineering, Cleveland Clinic, Lerner Research Institute, Cleveland, OH, United States; ^2^Cleveland Clinic, Neurological Institute, Center for Neurological Restoration, Cleveland, OH, United States

**Keywords:** Parkinson’s disease, technology enablement, technology integration, healthcare transformation, clinical integration

## Abstract

Utilizing technology to precisely quantify Parkinson’s disease motor symptoms has evolved over the past 50 years from single point in time assessments using traditional biomechanical approaches to continuous monitoring of performance with wearables. Despite advances in the precision, usability, availability and affordability of technology, the “gold standard” for assessing Parkinson’s motor symptoms continues to be a subjective clinical assessment as none of these technologies have been fully integrated into routine clinical care of Parkinson’s disease patients. To facilitate the integration of technology into routine clinical care, the Develop with Clinical Intent (DCI) model was created. The DCI model takes a unique approach to the development and integration of technology into clinical practice by focusing on the clinical problem to be solved by technology rather than focusing on the technology and then contemplating how it could be integrated into clinical care. The DCI model was successfully used to develop the Parkinson’s disease *Waiting Room of the Future (WROTF)* within the Center for Neurological Restoration at the Cleveland Clinic. Within the WROTF, Parkinson’s disease patients complete the self-directed PD-Optimize application on an iPad. The PD-Optimize platform contains cognitive and motor assessments to quantify PD symptoms that are difficult and time-consuming to evaluate clinically. PD-Optimize is completed by the patient prior to their medical appointment and the results are immediately integrated into the electronic health record for discussion with the movement disorder neurologist. Insights from the clinical use of PD-Optimize has spurred the development of a virtual reality technology to evaluate instrumental activities of daily living in PD patients. This new technology will undergo rigorous assessment and validation as dictated by the DCI model. The DCI model is intended to serve as a health enablement roadmap to formalize and accelerate the process of bringing the advantages of cutting-edge technology to those who could benefit the most: the patient.

## 1. Introduction

Over the past several decades there has been an explosion in the development of technology and the “internet of things” aimed at providing objective and quantitative outcomes to accelerate the detection and improve the treatment of Parkinson’s disease (PD) (1). Despite this explosion, the concept of using objective and quantitative measures to characterize PD symptoms and motor and non-motor function is not new. In the early 1950’s accelerometers were used to measure human gait (2) and in the 1970’s the possibility of using accelerometers to characterize human movement in athletes was realized (3). The pioneering studies of George Stelmach (4) and Erwin Montgomery (5) were some of the first to apply biomechanical methods to better understand the effects of PD on motor control and potentially aid in disease detection. However, after decades of development and potential promise of using objective, quantitative outcomes from these and other technologies to enhance patient care, the gold standard of PD evaluation remains a subjective clinical scale. The goal of this paper is to introduce a cohesive model of technology development and clinical integration that we have used to effectively transition technology from the peak of inflated expectations through the trough of disillusionment and eventually to the plateau of productivity for the benefit of patient care and scientific advancement.

**Figure 1:**
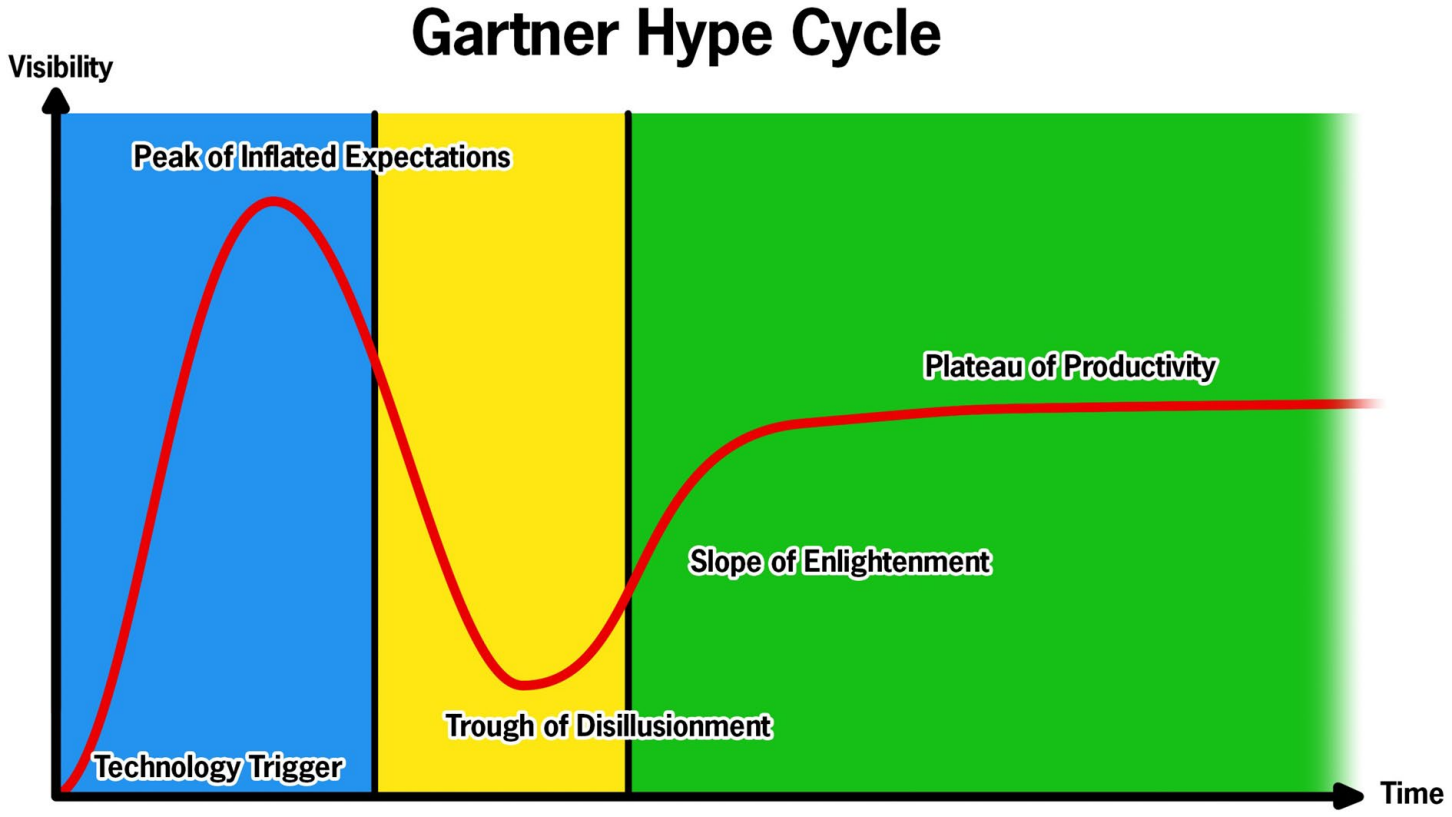
Representation of the Gartner Hype Cycle for technology with the corresponding components of the Develop with Clinical Intent (DCI) model. The blue represents the Technology Development and Validation Phase of the DCI model; the yellow represents the Clinical Viability and Design Iteration Phase; and green represents the Full-scale Clinical Integration.

Gartner, Inc. (Stamford, CT), the advisory and information technology company, proposed a hype cycle “model” that characterizes technology adoption (Figure 1). The hype cycle consists of five phases: technology trigger, peak of inflated expectations, trough of disillusionment, slope of enlightenment, and plateau of productivity. The hype cycle is intended to conceptualize the maturity of technology and its adoption. While not a perfect model, it appropriately contextualizes the use of technology in evaluating PD motor and non-motor performance. We take the position that technology intended to aid clinical practice in PD has cycled between the first three phases of this hype cycle: an emerging technology triggers an explosion of enthusiasm and validation studies and maybe even a few case series studies are published and then that technology tumbles to its final resting place, the trough of disillusionment. Failure to integrate promising technology has stagnated the field of movement disorders neurology, visible by the continued reliance on the Unified Parkinson’s disease Rating Scale (UPDRS), originally developed in the 1980s (6).

**Figure 2:**
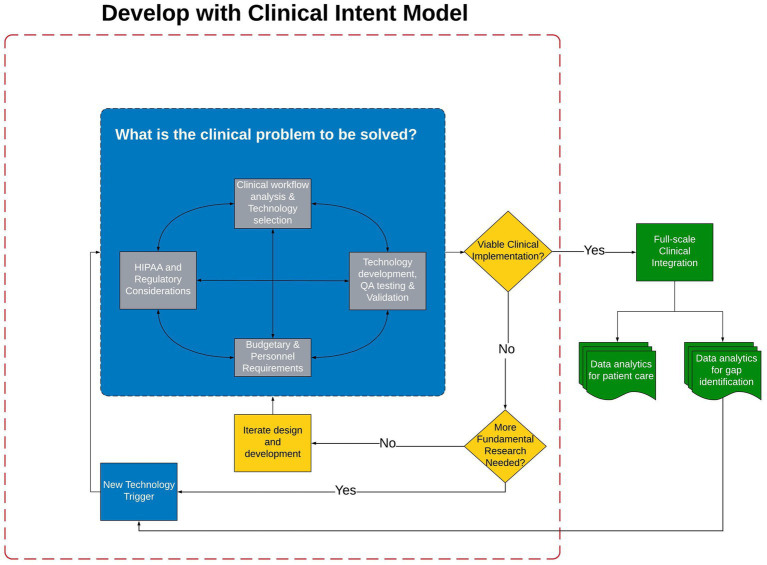
The Develop with Clinical Intent Model and decision-making points. Phase I, the blue section, represents the steps that must be considered during the initial phase of Technology Development and Validation. Once validation has occurred the technology is evaluated for Viability of Clinical Implementation in Phase II (represented by yellow decision points). Should the technology be viable for clinical integration, Full-scale Clinical Integration is Phase III (green section). HIPPA, Health Insurance Portability and Accountability Act; QA, Quality assurance.

The field of clinical neurology, movement disorders in particular, is filled with examples of technology developed to quantify a single, isolated PD symptom via accelerometer or other technologies (7–11). In a review of technology solutions for the quantification of PD motor and non-motor symptoms, only six of more than 500 technologies were deemed at a technology readiness level for the integration into clinical care (12); of those six, it is unclear if any have been integrated into routine clinical care. The inability to integrate into clinical workflows provides clear evidence the field must critically reassess the model of technology development to ensure the technology has the best chance to pass through the trough of disillusionment. Hence, the expert Movement Disorders Society panel continues to call for the development of technology platforms that can be integrated into clinical workflows (13). Previous technology often times is valid and reliable; however, the focus has been on technology development with little regard to feasibility of clinical integration (14, 15). If the true value of technological approaches to quantifying motor and non-motor aspects of PD are to be realized, a fundamental shift in the approach to technology development and integration is necessary. We have created and successfully utilized the Develop with Clinical Intent (DCI) model, shown in Figure 2. Central to this model is that technology development is secondary to the clinical problem that the technology aims to solve.

The DCI model was used to guide the successful development and integration of the Multiple Sclerosis Performance Test (MSPT) mobile application (16). The MSPT application is the cornerstone to the multi-continent Multiple Sclerosis Partners Advancing Technology and Health Solutions (MS PATHS) which is the first example of a learning health system in MS (17). To date, more than 17,000 unique MS patients have completed the MSPT application as part of standard of care; approximately 88 percent of these patients have multiple assessments over time which has resulted in more than 93,000 quantitative assessments of motor and non-motor function in approximately six years. Data from the MSPT application has informed and augmented the care of the individual patients, enhanced care of MS patients from a population health perspective (18–21), and reduced provider documentation time in the electronic health record (22). Our experience in developing the MSPT application and involvement with the MS PATHS initiative was leveraged to create the PD-Optimize application for deployment into the Parkinson’s disease Waiting Room of the Future for PD (PD-WROTF).

## 2. Operationalizing the Develop with Clinical Intent model to create the Parkinson’s disease waiting room of the future

Building the PD-Optimize application and PD-WROTF was initiated in 2019; following pandemic related delays it was integrated into clinical practice and the Center for Neurological Restoration at the Cleveland Clinic in 2021. The remainder of this manuscript will detail the DCI model and processes, experience with the PD-WROTF and how analytics and clinical experience are shaping the development of new technology to better understand the effects of PD on non-motor performance and completion of instrumental activities of daily living (IADLs). It is envisioned that the DCI model serve as a roadmap for the development and integration of technology into routine clinical practice. The DCI model and decision-making process has three phases: (I) Technology Development and Validation (Figure 2, blue section), (II) Clinical Viability and Design Iteration (Figure 2, yellow section) and (III) Full-scale Clinical Integration (Figure 2, green section).

### 2.1. Phase I: technology development and validation

The critical first step of the DCI model is to bring providers, patients, engineers, IT professionals and data scientists together to clearly identify: *What is the clinical problem to be solved?* For PD, the clinical problem is well-known and has been expressed over multiple decades: how can one comprehensively and objectively quantify PD motor and non-motor symptoms for use in the long-term tracking of disease progression to optimize the clinical management of PD patients? While this clinical problem may be rather obvious to providers or scientists who are immersed in PD clinical care or investigation, it is likely not evident to those who will be developing, testing and evaluating, integrating and eventually using the technology. Having critical and open conversations with experts in their respective fields will ensure all important knowledge and experience will be evaluated and weighed to ensure that the proposed technology is the most suitable, scalable and sustainable to solve the clinical problem.

While the problem of quantifying PD motor and non-motor function is well-known to PD providers and researchers, software developers and IT professionals are likely not familiar with the problem. The *Clinical Workflow Analysis and Technology Selection* is an important early step that must have representation from all stakeholders. The active participation of all parties is necessary to ensure all teams have a clear understanding of the problem and how they can leverage their respective expertise to identify a technology solution that can be scaled and sustained. It is necessary to engage engineers, software developers and IT specialists, including those affiliated at the enterprise level, in this initial phase as these preliminary discussions reinforce the concept that the project is not about the technology, rather that technology should enable the practice of better and more efficient medicine to benefit the patient. *Technology Selection* discussions between clinicians, researchers, and engineers are critical in this early phase of the DCI model as identifying the most appropriate technology to address the problem is critical. Our approach to the *Technology Selection* phase is to empower the engineers and software developers to lead this phase as they are familiar with the strengths and weaknesses of a given technology. If this phase is executed properly, the software and hardware developers will often propose technology that is typically “cutting edge,” but may not be “bleeding edge.” This phase takes time, commitment and discipline. Clinicians typically want to implement tomorrow, researchers want to use bleeding edge technology and if developers and IT are not actively participating in this phase, they want to finish the application and move to the next project.

Once the technology has been agreed upon, the *Clinical Workflow Analysis* phase is undertaken. The goal of this phase is to immerse the developers and engineers with all providers in the clinic to understand the current clinical workflow. Ideally, these teams will spend multiple days together initially and make frequent visits to the clinical setting during the technology development phase. Understanding the existing patient flow and how information is transmitted or not transmitted between nurses, physicians and patients will provide insight into the optimal integration points or may reveal that the technology can only be used if there is a reimagining of the clinical workflow.

A necessary precursor to the adoption of technology into the clinical workflow is provider trust of the data generated by the new technology. Providers must trust that the technology is accurate, reliable and provides data that will enhance treatment. During the *Technology Development, Quality Assurance (QA) Testing, and Validation Testing*, trust is established between the clinical team members, the technology team members, and the technology itself. We contend that the most appropriate technology validation study design is one in which outcomes from the proposed technology are compared to a gold standard biomechanical or non-motor outcome if possible. Notably, it is contended that the correlation of a measure of motor function to a clinical rating within the MDS-UPDRS III is not a gold standard comparison of motor performance. While MDS-UPDRS III items are the clinical gold standard for clinical use, they are not objective and lack resolution and a degree of quantification to serve as the best validation comparison to a new technology (23). If the outcome from the technology simply correlates with a subjective clinical rating, one must question if that is the best use of technology as providers will likely reject the technology as it is not providing new information that they could not derive from the traditional clinical assessment. Following rigorous validation, an analysis to understand how the outcomes of the technology may be related to clinical ratings to facilitate clinical understanding and adoption is recommended.

Once the team is aligned on the clinical problem, understands clinical workflow challenges and opportunities and validation strategy is established, the inevitable and unavoidable *Budgetary and Personnel Requirements* discussion must occur. Each institution will have resources unique to them that will shape these discussions. Seeking philanthropy for the support of a clinical technology integration project may an effective strategy or, as in the case of the MS PATHS, collaboration with a pharmaceutical partner. It is important to consider not only the technical development and validation of the potential technology, but also ongoing support in terms of maintenance, data storage, hardware and cost of integrating data into the electronic health record (EHR). The PD-Optimize and PD-WROTF were largely supported by philanthropic support.

Although it may seem premature during the initial development and validation phase, it is valuable to engage and contemplate the current *HIPPA and Regulatory Considerations* and future regulatory claims of the technology. During the initial phases of development, the most important activity is keeping and maintaining comprehensive documentation. Notably, documenting user requirements, technical requirements, software versioning, hardware versioning and quality assurance testing will save time and effort when deploying to the clinic as this information will be requested by enterprise IT and EHR teams. Further, any documentation of human factors testing, no matter how informal, will be important should one decide to pursue regulatory approval in the future.

### 2.2. Phase II: clinical viability and design iteration

Once the technology is at the point of moving out of the development phase, a piloting of the solution is necessary (Figure 2, yellow section). It is recommended that the beta version of the software/hardware should be tested within the clinical practice of one of the providers engaged in Phase I of the DCI model to determine if the technology is: *Viable for Clinical Implementation.* If the technology is not mature enough to meet the viability criteria (e.g., does it work reliably, is the user interface appropriate, can patients complete, are the data outputs valid and reliable, etc.), then one must determine if more fundamental research or validation or development is necessary. As shown in Figure 2, if the answer to this question is *No*, the technology should re-enter the development cycle and the stakeholders iterate on design or user interface or whatever factor(s) has been identified as a barrier to clinical integration. If more fundamental research is needed, one must critically evaluate if the current technology is capable of addressing the question or if alternative or new technology should be considered.

### 2.3. Phase III: full-scale clinical integration

If the technology is determined to meet clinical viability requirements, *Full-scale Clinical Integration* follows (Figure 2, green section). Prior to this implementation, the technology must be industrialized or hardened to ensure it is reliable and does not require a full-time engineer to monitor and troubleshoot. It is at this point the initial time and effort spent with the enterprise IT and EHR groups will pay dividends as their approval is necessary for the introduction of a new technology and for the integration of outcomes into the EHR. The transition from clinical viability to full-scale clinical integration is tenuous and failure to plan for this transition from Day 1 increases the probability of the technology getting stuck in the trough of disillusionment. An enterprise approved plan for continuous support and maintenance of the technology must be contemplated and agreed upon prior to clinical implementation. Clear communication with enterprise IT to clearly understand who will be responsible for perpetual support and maintenance is critical. It is also important to facilitate continuous communication between the data analytics team, IT team and medical providers after clinical integration as it relates to the display of data outcomes and their formal analyses. While many EHRs are able to ingest data from mobile devices or external sources, the graphing and visual display capabilities of the EHR are far from that of typical analytic or statistical software packages. In our experience, integration with EHRs should be done as close to natively as possible. Native integration typically limits data exchange to raw data; however, it lends itself to reliability and good clinical utilization as providers are able to minimize the number of clicks between screens or data sources.

The successful clinical integration and utilization of the technology is a tremendous accomplishment: however, in order to sustain use of the technology proper utilization of these data are necessary. One must remember that patient appointments are likely a little longer to complete the technology assessment. It is critical that patients are informed of these changes in clinical workflow prior to their appointment so they know what to expect and can plan to bring their eyeglasses or hearing aid. By asking patients to complete these tests prior to their appointment there is an implied importance of these assessments. It is imperative that the provider review these data with the patient as part of their clinical visit. Failure to review the data with the patient will result in the patient feeling these data are not important and they are likely to not complete in future visits (24).

Finally, clinical interpretation and data analytics should be coupled to identify new questions and potentially trigger the revision of the technology or possibly trigger the development of new technology. This encourages continued use and improvement of the DCI model.

## 3. Experience with the PD waiting room of the future (PD-WROTF)

The Center for Neurological Restoration (CNR) is a PD Center of Excellence that serves Northeast Ohio. Across all of its locations, the CNR examines and treats over 10,000 unique patients annually from across the globe. The DCI model was used to create the PD-WROTF which aimed to gather objective and quantitative data to better understand, track and treat the motor and non-motor effects of PD. The PD-WROTF was introduced into clinical practice in two stages. The first stage was in late 2019. To evaluate the acceptance of using technology, PD patients completed quality of life and symptom severity questionnaires via an iPad after checking in for their appointment. These questionnaires were completed by the patient in the waiting area. Information from the questionnaires was then automatically uploaded to a HIPPA compliant cloud, hence the importance of getting enterprise IT and cybersecurity engaged early, and automatically integrated into a flowsheet within the EHR. The provider was able to review this information and discuss with the patient at that visit. The collection of these patient reported outcomes served as a “soft launch” to determine if technology could be successfully implemented into a patient visit without disrupting the overall clinical workflow and gather patient and clinician feedback.

Early in the *Technology Selection* and *Technology Development* discussions, there were valid concerns from providers, software developers and IT staff that individuals with PD tend to be older and exhibit physical limitations such as bradykinesia, tremor or cognitive impairments that would result in poor assessment compliance and high abandonment rates. To mitigate these concerns, a group of PD patients were engaged in the development process and assisted in developing instructions for the test that were understandable and that the user interface and experience contemplated the effects of potential motor and non-motor dysfunction. Early engagement of the end user has been critical in ensuring the assessment modules are completed as intended and that the outcomes are measuring the function of interest.

Another key concern from clinicians was the amount of oversite that would be required to ensure the patients were completing the modules as intended. During the *Clinical Workflow Analysis and Technology Selection,* the clinicians voiced the importance of self-administered modules to minimize staffing requirements and maximize workflow efficiency. The clinical and software teams addressed this need through the development of self-administered modules, ultimately resulting in one medical assistant overseeing as many as five PD patients simultaneously completing the PD-Optimize app. The cognitive modules include a practice session to ensure the patient understands the task. Algorithms were created during the *Technology Development, Quality Assurance (QA) Testing, and Validation Testing* to ensure understanding; if the patient makes too many errors on the practice portion, they are re-directed back to the instructions. The algorithms went through a rigorous quality assurance testing. This process ensured full understanding of the task prior to assessment initiation. Furthermore, the clinicians felt empowered that their clinical integration concerns had been heard and collaboratively addressed in the initial phase of the project.

**Figure 3:**
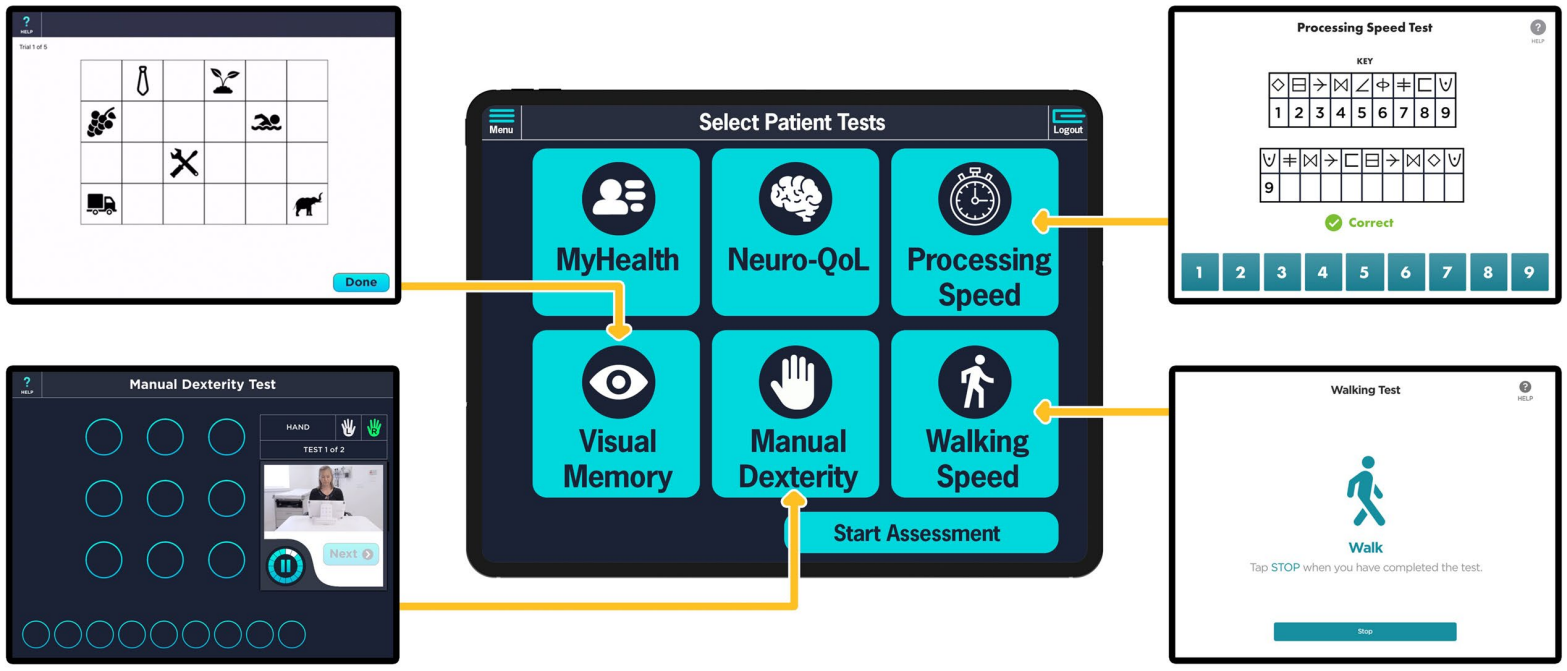
PD-Optimize modules presented to PD patients in the Parkinson’s disease – Waiting Room of the Future (PD-WROTF). The middle column illustrates the home page initially presented to the patient showing the modules that will be completed. The modules are self-administered at the patient’s own pace with the ability to repeat instructions to ensure understanding. The Medical Assistant has the option to de-select a given module if a patient would not be able to complete (e.g., a patient who is non-ambulatory may not complete the Walking Speed module). Screenshots of the Visual Memory Test, Manual Dexterity Test, Processing Speed Test, and the Walking Speed Test are displayed in the left and right columns. The MyHealth questionnaire asks the question “What is most important item(s) you want to discuss with your care team today?” The Neuro-QoL is a patient-reported quality of life metric for adults with neurological disease.

Based on the success of this initial launch and with appropriate adaptions from clinician and patient feedback, the full suite of cognitive and motor assessment modules of PD-Optimize was developed and integrated into CNR clinical workflow for all PD patients. In 2021, four assessment modules, screenshot shown in Figure 3, and the MyHealth patient demographics questionnaire were completed and incorporated into the clinical workflow. Similar to the MS PATHS, assessment modules are delivered to the patient via iPad. Two motor and two non-motor modules are self-administered by the patient. Upper extremity function is evaluated with the Manual Dexterity Test (16, 22, 25), an electronically enabled version of the Nine-Hole Peg Test (26, 27). Lower extremity performance is monitored using the Walking Speed Test, an electronic adaptation of the 10 meter walk test (28). From a non-motor perspective, two validated and normed assessments of cognition as well as a quality of life assessment are gathered. Information processing is evaluated using the Processing Speed Test (PST) (29–31) adapted from the Symbol Digit Modalities Test (32). The Visual Memory Test (VMT) evaluates episodic memory and delayed memory (31). The Quality of Life in Neurological Disorders (Neuro-QoL) is assessed as a patient-reported quality of life metric for adults with neurological disease (33). Based on our experience with implementation of the MSPT application (34), an important aspect of engaging patients in using technology for the collection of objective and quantitative data by completing assessments they are likely unfamiliar with is that the technology must serve their immediate needs as well. To address this need, patients are asked one open text response question in the MyHealth module: “*What is most important item(s) you want to discuss with your care team today?”* The response to this question is automatically populated at the top of the patient’s chart within the EHR and it is the first information shown to the clinician. Querying the patient about their most important concern has facilitated a more focused clinical visit for the patient and allows the provider to quickly see which patients on their schedule may have additional needs and make preparations in advance such as allocating more time to an encounter or further investigation into the patient’s chart and data prior to the encounter. Having providers engage patients around this question often facilitates a transition to a discussion about the objective data gathered. As we have demonstrated in MS-PATHS, these data bring the patient and provider together as patient’s report they are now both “speaking the same language” (22, 34).

## 4. Patient and data workflow

**Figure 4:**
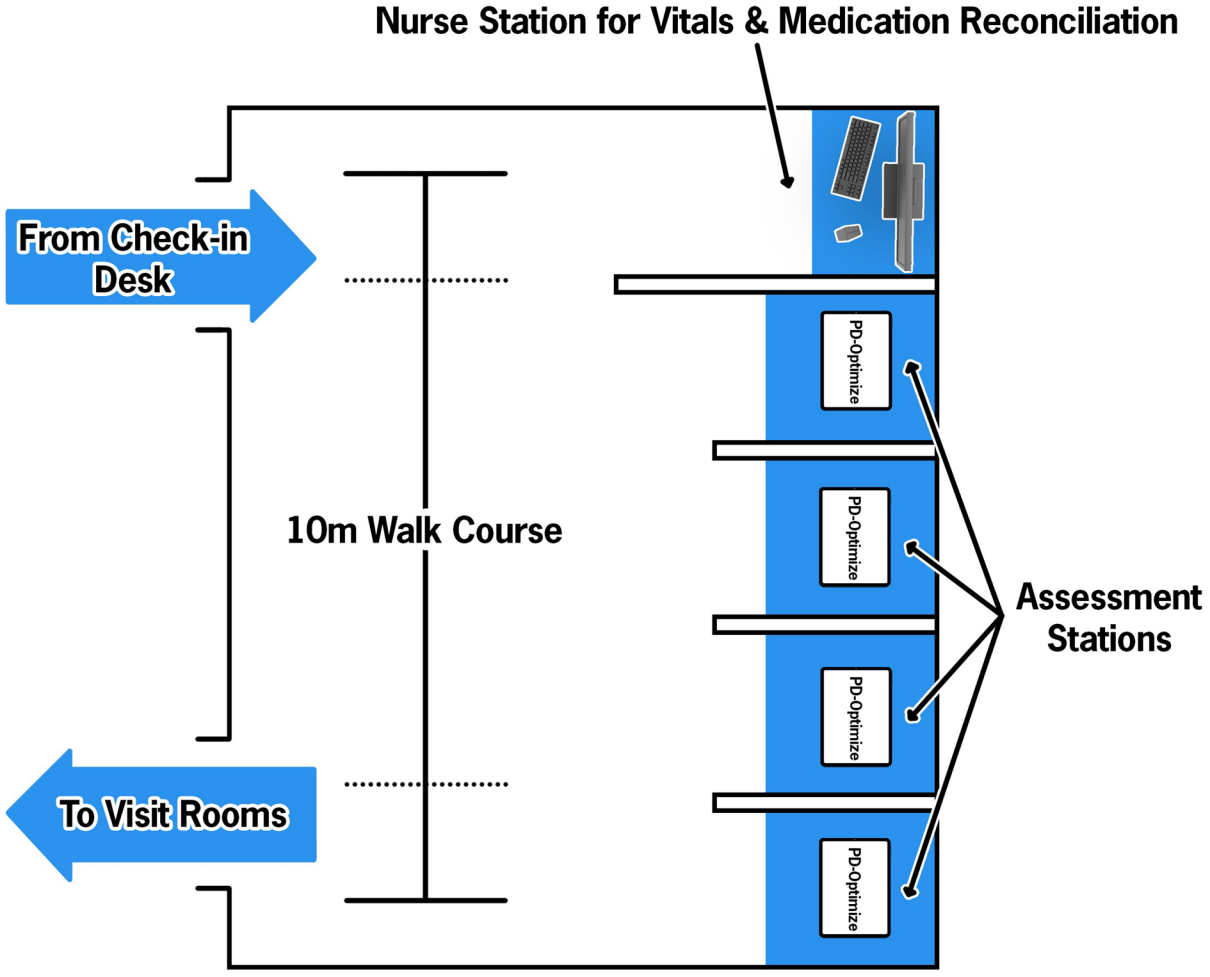
Parkinson’s disease-Waiting Room of the Future (PD-WROTF) at the Cleveland Clinic Center for Neurological Restoration. Following check-in at the front desk, the patient immediately enters the WROTF where they are greeted by a Medical Assistant (MA). The MA performs a standard vital sign assessment and medication reconciliation. The patient then proceeds to one of four assessment stations where an ITD-managed iPad is housed. The MA selects the patient’s name from the daily list and ensures the volume is appropriate via disposable headphones. Through the use of auditory and visual instructions, the patient progresses through each module of PD-Optimize. The modules are all self-administered and the single MA oversees the entire room, including the vitals station. Following completion of PD-Optimize, all data are automatically uploaded to the electronic health record (EHR) and the patient is escorted to their exam room.

As illustrated in Figure 4, when a patient arrives to the CNR, they check in at the front desk like a typical appointment. A medical assistant (MA) escorts the patient to the PD-WROTF. Following standard vital sign collection, the MA selects the patient from the schedule on the iPad and gives the iPad to the patient for them to complete the assessment modules. The MyHealth demographics questionnaire confirms identify from the patient, gathers demographics and instructs the patient on how to complete each assessment and ensures the patient can perform basic tasks on the iPad. The patient then completes the four cognitive and motor assessments, quality of life and demographic modules and returns the iPad to the MA. The patient is then taken to an exam room and proceeds with the medical appointment. Results from PD-Optimize are immediately available to the provider in the EHR for review, discussion with the patient and automatically populate clinical notes.

**Figure 5:**
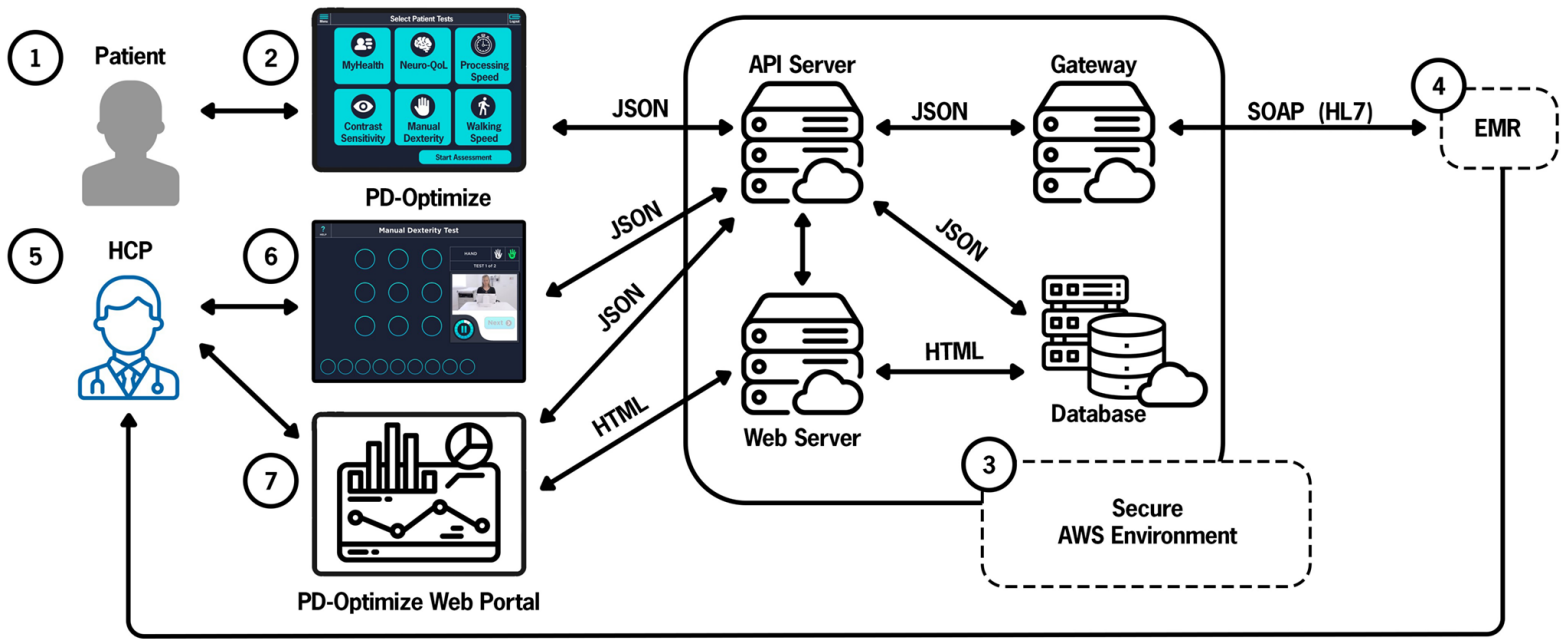
PD-Optimize cloud structure and data flow. Patient (1) inputs data using PD-Optimize application (2) and files are instantaneously uploaded to the PD-Optimize cloud, a HIPAA compliant, secure AWS environment (3). Files are transferred in JavaScript Object Notation (JSON) format. Files can be transferred to a web server, a cloud-based database or exported via a gateway to allow integration into the medical record (4). Outside of the medical record, the health care professional (5) can access patient data via the PD-Optimize application (6) or a secure web portal (7). API application programming interface, EMR electronic medical record, AWS Amazon Web Services, HCP health care professional, HIPAA Health Insurance Portability and Accountability Act, HL7 Health Level-7, SOAP Simple Object Access Protocol.

In addition to the PD-Optimize application on the iPad, two servers support the application. The cloud structure and data flow are shown in Figure 5. The architecture was discussed and agreed upon between the development team, cybersecurity and enterprise IT early in the development process. While these initial meetings seemed premature, they were critical in informing the security features that the application had to adhere to and the approach to encrypting the data when transmitting to the cloud and EHR. Briefly, the cloud-based PD-Optimize server, within the Amazon Web Services (AWS) environment stores all incoming data and outgoing assessment results. The iPad application communicates directly with the PD-Optimize server to retrieve information such as the CNR’s daily schedule and patient demographics, and to send assessment results to the PD-Optimize server to be stored and sent to the EHR. The second server, the Gateway, communicates bidirectionally with Cleveland Clinic’s EHR.

The PD-Optimize server communicates with the iPad to display the CNR’s daily schedule to the MA so they can select the correct patient before giving the iPad to the patient. To retrieve data from the EHR, the PD-Optimize server requests data from the Gateway, which in turn requests data from EHR’s Interconnect platform through an HTTPS web service. Interconnect uses a custom protocol to securely communicate directly with the primary EHR database. Next, the data from the EHR is transformed into a JSON file and returns the data in response to the gateway’s web service call. The gateway then returns the data to the PD-Optimize server. When an iPad requests the schedule from the PD-Optimize server, it returns the most recent EHR data from the gateway server.

When a patient completes the assessment modules, the PD-Optimize application immediately uploads the results as JSON to a URL on the PD-Optimize server. The PD-Optimize application stores the assessment results to a secure research database to make the data easily available and preserved. The PD-Optimize server also sends the data to the gateway server, which sends the data to the EHR for storage in the EHR database as flowsheet data. Because the assessment results are stored in the EHR in real-time, providers can view the results in the EHR during the visit, compare with past results, and include the most recent results in their notes.

As of January 1, 2023 more than 2,000 unique patients have completed the PD-Optimize application within the WROTF. Nearly 300 patients have completed PD-Optimize at two or more clinical visits. On average, 20 min is required to complete the assessments, including demographics confirmation.

We are currently assessing the response of dopaminergic therapy on bradyphrenia by comparing PST before and after a change in medication. Considering the PD WROTF is still in its nascence, much of its potential in better understanding PD and the development of a biomechanical biomarker will emerge as more patients complete the assessments over repeated clinical visits. Nevertheless, from data collected to date we have surmised that PD results in specific cognitive issues. Specifically, deficits in processing speed and executive function have been observed at rates greater than clinicians anticipated and have led clinicians to question if cognitive deficits observed in the PD-Optimize application map onto difficulties performing instrumental activities of daily living (IADL) in a ‘real-world’ environment. Fundamentally, these initial insights in cognitive functioning have served as a technology trigger in terms of better understanding how the PD-Optimize outcomes map onto PD patients’ performance of IADLs. These discussions between clinicians and researchers have cascaded a *New Technology Trigger* (Figure 2) that aims to leverage the capabilities of virtual reality (VR) to evaluate IADL performance in PD.

## 5. Peak of inflated expectations? Cleveland Clinic virtual reality shopping platform

Patients, providers, hospitals and regulatory bodies are increasingly interested in outcome measures that quantify the effects of PD motor and cognitive symptoms in meaningful daily actions (13, 35–37). Technological advances, like PD-Optimize, provide the opportunity to measure motor and cognitive symptoms for more precise and meaningful measures of PD symptoms. The assessment of IADLs are necessary to systematically evaluate the overall effectiveness of an intervention in a salient environment or determine the potential of an intervention to slow disease progression.

Cooking, crossing a busy street, getting groceries and driving a car (38) are common IADLs that may be compromised in PD patients. IADLs are necessary for independent living and community integration (39), and frequently require the simultaneous performance of two attention-demanding tasks (e.g., motor-cognitive, motor-motor or cognitive-cognitive) (40). It is not realistic to avoid dual-task conditions, as they are necessary to complete the vast majority of daily household and community activities (41, 42). Although dual-task declines associated with PD clearly impact IADL performance, traditional clinical motor evaluations (43–45) and neuropsychological tests (46, 47) are insufficient to evaluate IADLs as they parse cognitive and motor function into distinct components or constructs without consideration of their interplay. Innovative virtual reality technology provides a method of delivering ecologically valid digital content for the patient to interact with and quantifying those interactions using rigorous biomechanical measures.

**Figure 6:**
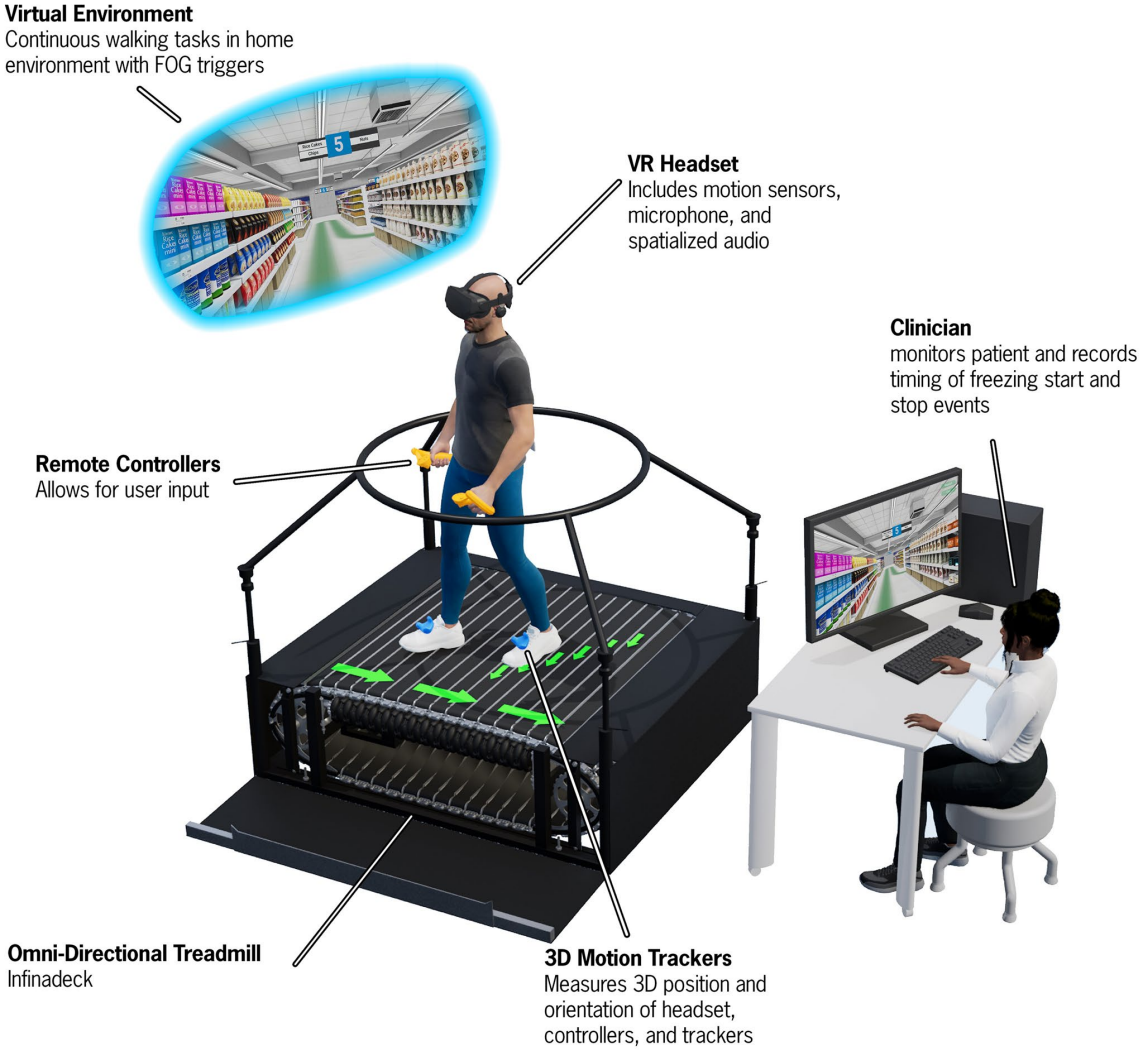
A depiction of the Cleveland Clinic-Virtual Reality Shopping (CC-VRS) platform. The patient wears a VR headset and physically walks on an omnidirectional treadmill to navigate through a virtual grocery store. The patient follows a green path and is required to gather five items along this path. Various obstacles (e.g., narrowing of aisles and avoiding spills) and other shoppers must be avoided while performing the CC-VRS. Biomechanical data are derived from position data provide by the 3D motion trackers on the feet and Remote Controllers to characterize gait and upper extremity prehension. A first-person view of the user is provided to the experimenter via the Control Computer and Monitor.

Based on feedback from providers utilizing the PD-WROTF technology, we identified a gap in the efficient, systematic and quantitative approach to quantifying PD IADL performance. We assembled a team of biomedical engineers, software developers, physical therapists, occupational therapists, and neurologists to create a virtual reality shopping task that had all of the key aspects of motor, cognitive and cognitive-motor components to understand how PD patients perform IADLs. As detailed previously (48), state of the art VR technology was combined with an omnidirectional treadmill which allowed PD patients to physically navigate a virtual grocery store. The Cleveland Clinic Virtual Reality Shopping (CC-VRS), shown in Figure 6, aims to objectively quantify the performance of IADLs in PD patients. The CC-VRS platform addresses the clinical gap by providing a standardized, systematic, objective and quantitative approach to characterizing IADL capabilities in older adults and those with neurological disease. Briefly, the participants complete a 3-min tutorial to ensure understanding of walking on an omnidirectional treadmill and hand trackers (used to display the list and retrieve objects), and to expose the participant to the VR grocery store environment. In order to advance to the CC-VRS assessment, participants must demonstrate proficiency (automatically and objectively measured by the application) in walking, viewing the grocery list, and selecting the item on the list in the tutorial. Once deemed proficient, the patient is progressed to two different CC-VRS scenarios. The Basic CC-VRS requires the patient to ambulate through a grocery store and select 3–5 items from their list. The Complex CC-VRS has the same requirements as the Basic and additionally the patient encounters motor challenges such as narrowed aisles and other shoppers along the path as well as cognitive challenges such as identifying the more cost-effective sale item. Based on preliminary usability testing and data, the Basic and Complex CC-VRS Scenarios can be completed in approximately 12–20 min total.

The CC-VRS is currently being used in two research projects aimed at: (1) identifying the neural signature underlying freezing of gait in advanced PD patients with deep brain stimulation systems and (2) validating performance on the CC-VRS in a group of young adults, older adults, and individuals with PD. These research projects have been critical in supporting the development of the VR technology and validation of outcomes relative to overground walking. We are currently evaluating the clinical viability of deploying this technology by conducting an initial pre-deployment study in a regional family health center. As part of this validation project, 400 healthy older adults will complete the CC-VRS as part of their annual Medicare Wellness Assessment. The outcomes of this project will provide valuable clinical experience and normative healthy older control data that can be used in better understanding the precise effects of PD and will inform whether the CC-VRS will be employed to all CNR PD patients or if a subset of the population would be more appropriate, such as those under consideration for deep brain stimulation as the CC-VRS provides and ecological assessment of dual-task functioning, which is known to be affected by deep brain stimulation (49).

## 6. Moving through the hype cycle

Technology continues to be developed at a dizzying pace, and health care settings continue to be slow to adopt and adapt this technology to better serve patients during routine clinical care. The DCI model outlines a potential path for technology development that is scalable and adaptable. Developing technology for clinical integration may at times feel like trying to untie a Gordian knot. Unfortunately, the DCI model does not have a secret Alexander the Great sword, however, the model should assist in the time, personnel, expense, outreach, and other resources necessary for meaningful technology development and integration. The DCI model is not a formula, rather it is intended to serve as a roadmap to work through the fluid process of integrating technology into clinical workflows. While the specifics may look different depending on factors such as patient population, healthcare system, and resources, the overarching principles are applicable to many sectors across the healthcare system. It should be acknowledged by all stakeholders that not all technologies will progress to clinical integration using the DCI model; some technologies will not be able to progress out of the peak of inflated expectations or the trough of disillusionment. This is expected, and even encouraged, to ensure the technologies that do advance to clinical integration have been rigorously evaluated and truly enhance the provider and patient experience.

There have been trials and tribulations in the implementation of the PD-WROTF. The group ownership of the project within the DCI model provided a strong sense of ownership in which the failures and success of the project were mourned and celebrated by the entire team. We have now reached a point where the technology has addressed a clinical problem and the patient-provider relationship has been strengthened.

## Data availability statement

The original contributions presented in the study are included in the article, further inquiries can be directed to the corresponding author.

## Author contributions

All authors listed have made a substantial, direct, and intellectual contribution to the work and approved it for publication.

## Funding

This manuscript was supported by the Edward and Barbara Bell Family Endowed Chair (JA), the Michael J. Fox Foundation for Parkinson’s Research (MJFF-020020), and the National Institute of Health (R01NS129115). The content is solely the responsibility of the authors and does not reflect the views of any of the funding sources.

## Conflict of interest

JA and DS have authored IP related to the PD-Optimize modules; JA and AR have authored IP related to the CC-VRS modules.

The remaining authors declare that the research was conducted in the absence of any commercial or financial relationships that could be construed as a potential conflict of interest.

## Publisher’s note

All claims expressed in this article are solely those of the authors and do not necessarily represent those of their affiliated organizations, or those of the publisher, the editors and the reviewers. Any product that may be evaluated in this article, or claim that may be made by its manufacturer, is not guaranteed or endorsed by the publisher.
